# Using linezolid as a substitute for the injectable in case of ototoxicity is safer and as effective as all-oral treatment for rifampicin-resistant TB

**DOI:** 10.5588/ijtldopen.25.0151

**Published:** 2025-08-13

**Authors:** M.B. Souleymane, T. Decroo, A. Soumana, I.M. Lawan, A.-L.C. Aboubacar, A. Gagara-Issoufou, R.H. Moussa, A-A.A. Kabirou, I. Hamidou, S.H. Moussa, M. Adamou, E. Adehossi, S. Mamadou, B.C. de Jong, L. Rigouts, A. Piubello

**Affiliations:** ^1^ Damien Foundation, Niamey, Niger;; ^2^ University of Antwerp, Department of Biomedical Sciences, Antwerp, Belgium;; ^3^ Institute of Tropical Medicine, TB-HIV Unit, Antwerp, Belgium;; ^4^ National Tuberculosis Programme, Coordination, Niamey, Niger;; ^5^ Hopital National Amirou Boubarcar Diallo, Service pneumo-phtysiologie, Niamey, Niger;; ^6^ Université Abdou Moumouni de Niamey, Faculté des Sciences de la Santé, Niamey, Niger;; ^7^ Centre Hospitalier Régional de Tahoua, CAT, Tahoua, Niger;; ^8^ Centre Hospitalier Régional de Maradi, CAT, Maradi, Niger;; ^9^ Université André Salifou de Zinder, Faculté des Sciences de la Santé, Zinder, Niger;; ^10^ Hopital National de Zinder, Service pneumo-phtysiologie, Zinder, Niger;; ^11^ Ministère de la Santé Publique, de la Population et des Affaires Sociales, Direction Générale de la Santé Publique, Niamey, Niger;; ^12^ Institute of Tropical Medicine, Mycobacteriology Unit, Antwerp, Belgium;; ^13^ Damien Foundation, Department of DR-TB, Brussels, Belgium.

**Keywords:** **KEYS WORDS:** tuberculosis, second-line injectable drug, bedaquiline, adverse events, Niger

## Abstract

**BACKGROUND:**

WHO recommends all-oral bedaquiline (BDQ) and linezolid (LZD)-containing regimens for rifampicin-resistant TB (RR-TB). In Niger, high cure rates were achieved using an adaptive short treatment regimen (aSTR) with a second-line injectable drug (SLID) and LZD, where LZD replaced the SLID in case of any ototoxicity detected on monthly audiometry. In 2020, WHO recommended a short oral BDQ/LZD regimen (oSTR). However, the success reported for oSTR was lower than for aSTR in Niger. The ‘SHOrt ORal Treatment’ trial therefore compared the safety and efficacy between aSTR and oSTR in Niger.

**METHODS:**

In this pragmatic clinical trial, patients with fluoroquinolone-susceptible RR-TB were assigned by alternate months to aSTR or oSTR. Regression models estimated the association between regimen and safety (grade 3-4 adverse events [AEs]) and efficacy (excluding loss to follow-up).

**RESULTS:**

Between 2021–2022, 158 RR-TB patients were included, 80 on oSTR and 78 on aSTR. Overall, 34 patients experienced 43 grade 3–4 AEs (anaemia: 15, neurotoxicity: 11, vomiting: 8, hepatitis: 7, arthralgia: 1, QTc prolongation: 1). Grade 3–4 AEs occurred in 26/80 (32.5 %) on oSTR versus 8/78 (10.3%) on aSTR, with anaemia, neurotoxicity and arthralgia being significantly higher in the oSTR group. Ototoxicity and nephrotoxicity appeared more frequently during the aSTR, but none evolved to grade 3. Patients treated with oSTR had a 3-fold increase in grade 3–4 AE (aHR 3.04;95% CI:1.36–6.80). End-of-treatment success was similar for oSTR compared to aSTR.

**CONCLUSION:**

aSTR was safer than oSTR and both approaches had a similar treatment efficacy.

WHO recommends all-oral shorter regimens containing new and repurposed drugs, such as bedaquiline (BDQ) and linezolid (LZD) for the treatment of rifampicin-resistant TB (RR-TB). Different all-oral regimens reduced the risk of mortality.^1–3^ Most all-oral BDQ-containing regimens also include LZD, the drug most frequently causing adverse events (AEs),^4^ including neurological and haematological AEs.^5–8^ The incidence of severe LZD-related AEs ranges from 10 to 40%, depending on dosage, duration and patient characteristics.^5,9^ In 35% of patients, these AEs can lead to permanent interruption of LZD or the entire treatment regimen.^1,9^

In Niger, over the past decade, an adaptive short treatment regimen (aSTR) containing a second-line injectable drug (SLID) achieved a high cure rate (> 80%). SLID was replaced with LZD if monthly audiometry detected any grade of hearing loss at baseline or ototoxicity during treatment.^10–12^ BDQ was safeguarded for those in need of a second RR-TB treatment. This was done within a well-established active TB drug safety monitoring and management (aDSM) framework.

In 2020, the WHO-recommended all-oral BDQ/LZD regimen (9–11 months) had a 73% success rate in South Africa.^4^ Replacing Niger’s aSTR with this all-oral STR would only be justified if robust evidence proves superior safety or efficacy. We therefore conducted a clinical trial, SHOORT (SHOrt ORal Treatment, PACTR202203645724919), with monthly alternating assignment to either a 9-month all-oral short treatment regimen (oSTR), with LZD throughout the intensive phase, or aSTR. Both regimens were compared in terms of safety and efficacy.^13^

## METHODS

We conducted a pragmatic clinical trial with monthly alternating regimens, involving all four facilities treating RR-TB patients in Niger. We used a single random draw; a coin toss before enrolment of the first patient determined the regimen to be used in even months and odd months of RR-TB diagnosis. Patients diagnosed with RR-TB in odd-numbered months were assigned to the oSTR, while those diagnosed in even-numbered months received aSTR.^13^ Due to the constraints of routine care, individual patient randomization was not feasible, and randomization at facility level would not overcome bias given that only four facilities participated. Month of RR-TB diagnosis determined treatment allocation. It was considered a random event due to the absence of known seasonal variation. Therefore, intra-cluster correlation (clusters defined by month of RR-TB diagnosis) was expected to be negligible. We opted for month of RR-TB diagnosis (shown on the printed drug susceptibility testing result) and not month of RR-TB treatment start, to avoid any effect of the clinician’s decision-making on regimen allocation.

Eligible participants included RR-TB patients diagnosed between 15 April 2021 and 31 October 2022 who initiated treatment; patients with fluoroquinolone-resistant TB and those previously treated for RR-TB were excluded. RR-TB was diagnosed using the Xpert MTB/RIF assay, while drug susceptibility testing (DST) for fluoroquinolones and SLIDs was performed using second-line line probe assays (LPA) and culture-based phenotypic DST on solid medium. The oSTR contained LZD (600 mg/day), high-dose isoniazid (Hh), prothionamide (PTO), high-dose levofloxacin (LFXh), BDQ, clofazimine (CFZ) and pyrazinamide (Z) for 4 months (6 months if delayed conversion on smear microscopy at treatment month 4), followed by 5 months of treatment with LFXh, BDQ, CFZ and Z (4-6 LZD-Hh-PTO-LFXh-BDQ-CFZ-Z/5 LFXh-BDQ-CFZ-Z). The aSTR contained a SLID (amikacin) or LZD, in case of any grade of hearing loss at baseline or ototoxicity detected on monthly audiometry during treatment (4-6 SLID-Hh-PTO-moxifloxacin [MFX]-CFZ-ethambutol [E]-Z/5 MFX-CFZ-E-Z, with LZD replacing the SLID in case of ototoxicity).

### Clinical follow-up and monitoring

All treated patients underwent directly observed treatment. aDSM for potential drug-related AEs included: (1) assessment of hearing loss at baseline followed by monthly pure-tone audiometry (frequencies between 250Hz−8000Hz) until the end of the intensive phase for patients on amikacin, (2) monthly complete blood count and haemoglobin for patients on LZD, paying particular attention to haematological toxicity, (3) renal function, visual and peripheral neuropathy tests (using colour discrimination test, and Brief Peripheral Neuropathy Screening) were assessed monthly for all patients, as were (4) electrocardiograms (ECG) at baseline, weeks 1 and 2 after the start of treatment, and then monthly during treatment.

AEs were recorded and classified by severity^14^ with grade 1 (mild), grade 2 (moderate), grade 3 (severe), grade 4 (life-threatening or permanently disabling). More specifically, haematological AE was monitored as follows: grade 1 = close monitoring, grade 2 = medical treatment, grade 3 = transfusion + reduction of LZD dose to 300 mg/day if smear microscopy converted to negative, and grade 4 = transfusion + LZD stop. The relatedness of AE was classified as likely related (AE emerges after a reasonable time from drug administration, resolves upon discontinuation, with no other clear cause) and definitively related (same as likely, with recurrence on re-challenge).

### Outcome definitions

The primary safety endpoint was the occurrence of any severe (grade 3–4) AE, likely or definitively related to prescribed TB drugs. WHO definitions were used for end-of-treatment outcomes: cured, treatment completion, death, failure and loss-to-follow-up.^15,16^ For the treatment efficacy endpoint, favourable outcomes were cured and treatment completion. Unfavourable outcomes were death and treatment failure. Patients lost to follow-up and those with initial fluoroquinolone resistance were excluded from the efficacy analysis.

### Statistical analysis

Associations between categorical variables were assessed using Chi-squared or Fisher’s exact tests (if the count was below 5). Frailty bivariable and multivariable Cox multivariable regression models accounting for the effect of assignment month were used to estimate the association between the treatment approach and the primary safety endpoint. Similarly, multilevel bivariable and multivariable logistic regression models were used for treatment efficacy (patients lost to follow-up were excluded). Adjustment was made for potentially confounding factors, such as age, gender, HIV status, body mass index (BMI), previous TB treatment, extent of disease on chest X-ray, microscopy bacillary load and TB clinic. Saturated models were constructed and simplified until only factors significantly associated with the outcome remained. The treatment approach was kept in the multivariable models regardless of the significance level. Analyses were done with Stata software (version 16.1, College Station, Texas).

### Ethical approval

The study protocol received ethical approval from the National Ethics Committee for Health Research of Niger (N°24/2021/CNERS of 08/04/21), the University of Antwerp ethics committee (N°21/07/112 of 01/03/21) and the Institute of Tropical Medicine of Antwerp ethics review board (N°1465/21 of 27/01/21). All participants provided informed consent.

## RESULTS

Countrywide, between 15 April 2021 to 31 October 2022, 174 patients were treated for RR-TB in Niger. Eleven patients were ineligible for our study because of locally diagnosed baseline fluoroquinolone-resistant RR-TB. Of the remaining 163 patients assigned to either oSTR or aSTR, 5 were excluded for being ineligible for the assigned regimen (Figure 1). A total of 158 patients were included in the analysis, 80 on oSTR and 78 on aSTR. Of 78 on aSTR, 36 (46.2%) were switched to LZD due to baseline (27) low-grade of hearing loss or emerging (9) low-grade ototoxicity. Of the 9 patients who switched from amikacin to LZD during treatment, 22.2% (2/9) used LZD for 1 month, 33.3% (3/9) for 2 months, and 44.5% (4/9) for 3 months. The median amikacin duration before the switch to LZD was 2 months (IQR:1–2).

**Figure.**
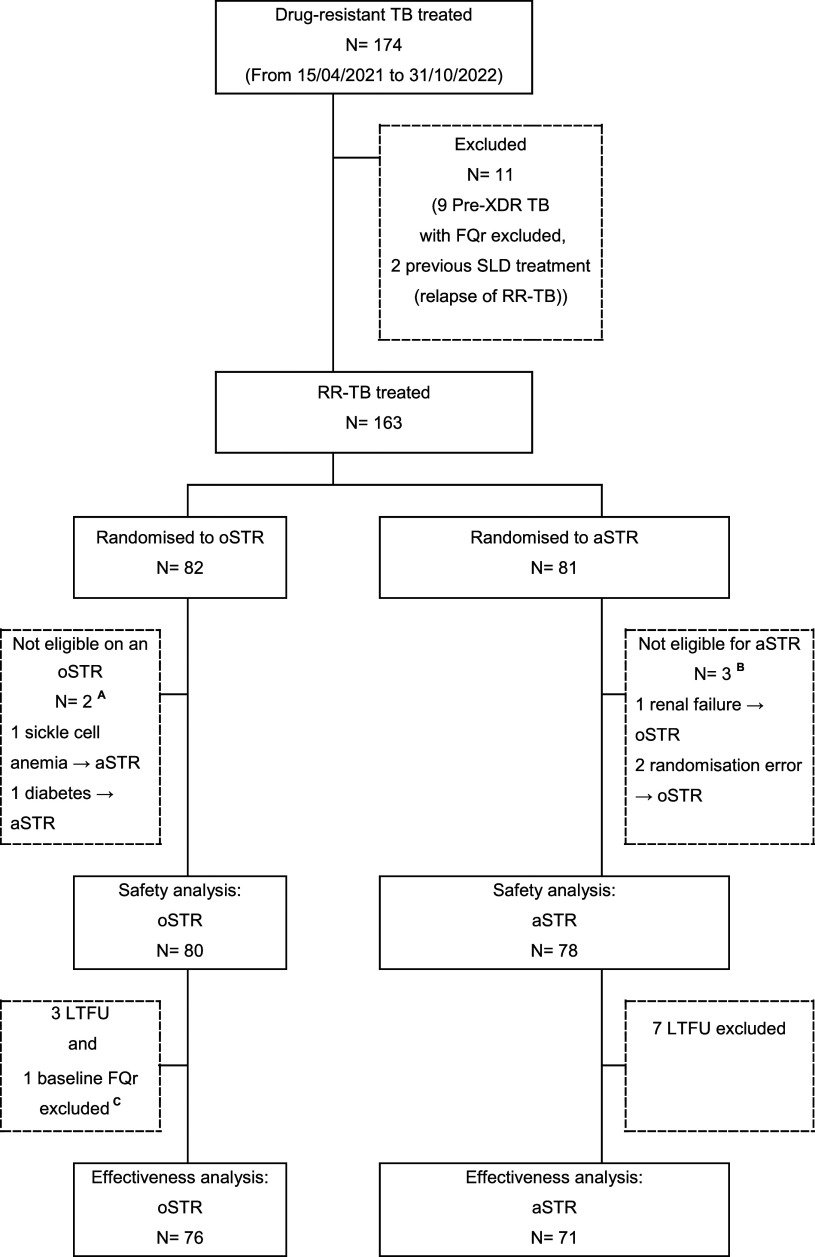
RRTB patients included in safety and efficacy analysis with exclusion of enrolment in study regimens.^**A**^Of two patients not eligible for All-Oral and treated with the aSTR, one was cured, and one died. ^**B**^Of three patients not eligible for the aSTR and treated with the oSTR approach, two were cured and one died. ^**C**^Excluded based on post-hoc testing of stored baseline samples, this patient was with baseline pre-XDR and failed the treatment on oSTR. Pre-XDR TB = pre-extensively drug-resistant TB; FQr = fluoroquinolone-resistant; RR TB = rifampicin-resistant TB; SLD = second-line drug; LTFU = lost to follow-up; aSTR: Niger treatment strategy with a second-line injectable drug (SLID)-containing short treatment regimen (STR), with linezolid replacing the SLID in case of any grade of ototoxicity on monthly audiometry; oSTR: all oral short treatment regimen with linezolid throughout the intensive phase.

Baseline demographic and bacteriological characteristics were similar between both arms (Table 1). Overall, 122 (77.2%) were male. The median age was 35 years (IQR: 28–47). Co-infection with HIV was present in 9 (5.8%) patients. Most participants had multiple cavities on chest X-ray (83; 52.5%), had received previous TB treatment (120; 75.9%), had a low BMI (median of 17.1 kg/m2; IQR:15.2–18.9), and a high (2+, 3+) bacillary load (100; 63.3%) on smear microscopy. Most were treated at the Niamey RR-TB facility (83; 52.5%).

**Table 1.** Baseline demographic and clinical characteristics.

Characteristics	Overall N = 158	oSTR N = 80	aSTR N = 78	p-value
**Sex**				**0.93**
Male	122 (77.2%)	62 (77.5%)	60 (76.9%)	
Female	36 (22.8%)	18 (22.5%)	18 (23.1%)	
**Age in years, median (IQR)**	35.0 (28.0, 47.0)	34.5 (27.0, 46.2)	35.5 (30.0, 47.8)	**0.27**
**Age category, years**				**0.97**
< 25	21 (13.3%)	11 (13.8%)	10 (12.8%)	
25 - < 50	102 (64.6%)	51 (63.7%)	51 (65.4%)	
≥ 50	35 (22.2%)	18 (22.5%)	17 (21.8%)	
**Body Mass Index, median (IQR)**	17.1 (15.2, 18.9)	17.3 (15.2, 19.2)	16.9 (15.4, 18.7)	**0.76**
**BMI category**				**0.88**
< 16.5	64 (40.5%)	31 (38.8%)	33 (42.3%)	
≥ 16.5 - < 18.5	45 (28.5%)	23 (28.7%)	22 (28.2%)	
≥ 18.5	49 (31.0%)	26 (32.5%)	23 (29.5%)	
**HIV test^A^**				**0.49**
Negative	147 (94.2%)	72 (92.3%)	75 (96.2%)	
Positive	9 (5.8%)	6 (7.7%)	3 (3.8%)	
**Type of TB**				**0.99**
Pulmonary	157 (99.4%)	79 (98.8%)	78 (100.0%)	
Extra-pulmonary	1 (0.6%)	1 (1.2%)	0 (0%)	
**Baseline microscopy bacillary load^B^**				**0.73**
High bacillary load (2+, 3+)	100 (63.3%)	50 (62.5%)	50 (64.1%)	
Low bacillary load (scanty, 1+)	44 (27.8%)	21 (26.2%)	23 (29.5%)	
Negative	13 (8.2%)	8 (10.0%)	5 (6.4%)	
Not performed**^C^**	1 (0.6%)	1 (1.3%)	0 (0.0%)	
**Prior TB treatment**				**0.16**
Previously treated for TB	120 (75.9%)	57 (71.3%)	63 (80.8%)	
None (“Primary” RR-TB)	38 (24.1%)	23 (28.7%)	15 (19.2%)	
**Treatment initiation delay from diagnosis, median (IQR)**	5 (3, 9)	5 (3, 8)	6 (3, 11)	**0.12**
**Chest radiography**				**0.49**
Bilateral cavitary lesion	83 (52.5%)	40 (50.0%)	43 (55.1%)	
Bilateral lesion	43 (27.2%)	22 (27.5%)	21 (26.9%)	
Unilateral & cavitary lesion	12 (7.6%)	7 (8.8%)	5 (6.4%)	
Unilateral lesion	6 (3.8%)	4 (5.0%)	2 (2.6%)	
Not performed	11 (7.0%)	4 (5.0%)	7 (9.0%)	
Normal	3 (1.9%)	3 (3.8%)	0 (0.0%)	
**RR-TB facility**				**0.05**
Niamey	83 (52.5%)	34 (42.5%)	49 (62.8%)	
Maradi	33 (20.9%)	21 (26.2%)	12 (15.4%)	
Tahoua	31 (19.6%)	17 (21.2%)	14 (17.9%)	
Zinder	11 (7.0%)	8 (10.0%)	3 (3.8%)	
**Month of conversion on sputum smear microscopy^D^**				**0.08**
Month 1	87 (55.4%)	52 (65.8%)	35 (44.9%)	
Month 2	35 (22.3%)	12 (15.2%)	23 (29.5%)	
Month 3	9 (5.7%)	4 (5.1%)	5 (6.4%)	
Month 4	3 (1.9%)	1 (1.3%)	2 (2.6%)	
Month 5	3 (1.9%)	1 (1.3%)	2 (2.6%)	
Month 6	2 (1.3%)	2 (2.5%)	0 (0.0%)	
Not converted^E^	18 (11.5%)	7 (8.9%)	11 (14.1%)	
**Baseline FQ molecular test^F^**				**0.42**
Wild type	122 (84.1%)	60 (81.1%)	62 (87.3%)	
Negative for MTB	22 (15.2%)	13 (17.6%)	9 (12.7%)	
Resistance	1 (0.7%)	1 (1.4%)	0 (0.0%)	
**Baseline SLID (AMK) molecular test^G^**				**0.43**
Wild type	126 (85.1%)	63 (82.9%)	63 (87.5%)	
Negative for MTB	22 (14.9%)	13 (17.1%)	9 (12.5%)	
**Baseline culture result^H^**				**0.33**
Positive	95 (66.0%)	44 (60.3%)	51 (71.8%)	
Negative	45 (31.3%)	27 (37.0%)	18 (25.4%)	
**FQ and SLID phenotypic DST results on positive cultures**				
***FQ DST* ^I^**				**0.46**
*Susceptible*	92 (9 8.9%)	42 (97.7%)	50 (100%)	
*Resistance*	1 (1.1%)	1 (2.3%)	0 (0%)	
***SLID DST* ^I^**				**0.47**
*Susceptible*	93 (98.9%)	43 (97.7%)	50 (100%)	
*Resistance*	1 (1.1%)	1 (2.3%)	0 (0.0%)	
**Treatment duration median (IQR), days**	273 (270, 277)	273 (271, 278)	273 (209, 276)	**0.44**

A 2 patients with undetermined HIV test results were excluded.

B Determined by sputum smear microscopy after Ziehl-Neelsen or auramine staining.

C Extrapulmonary TB (Not performed).

D Excluded: 1 extra-pulmonary TB.

E Not converted: 11 deaths and 6 lost to follow-up before conversion and 1 failure.

F Excluded: 13 not conclusive results (5 invalids, 8 not done).

G Excluded: 10 not conclusive results (2 invalids, 8 not done).

H Excluded: 18 inconclusive cultures results (13 not done, 3 non-tuberculosis mycobacteria and 2 contaminated cultures).

I Excluded: 2 inconclusive results (2 invalids).

RR = rifampicin-resistant TB; IQR = interquartile range; N = number; FQ = fluoroquinolone; SLID = second-line injectable drug; AMK = amikacin; DST = drug-susceptibility test; aSTR = Niger treatment strategy with a second-line injectable drug (SLID)-containing short treatment regimen, with linezolid replacing the SLID in case of any grade of ototoxicity on monthly audiometry; oSTR = all oral short treatment regimen with linezolid throughout the intensive phase.

### Primary analysis: treatment safety

Among 158 patients, AEs were reported in 88,6% of cases. A total of 34 patients (21.5%) experienced at least one grade 3–4 AE. The 34 patients had 43 grade 3–4 AEs (anaemia: 15, neurotoxicity: 11, vomiting: 8, hepatitis: 7, arthralgia: 1, QTc prolongation: 1, ototoxicity: 0). Grade 3–4 AE were significantly more frequent with oSTR: 26/80 (32.5%) versus 8/78 (10.3%) (p<0.001) – see Table 2. Of 8 patients with grade 3-4 AEs on aSTR, 6 had been switched to LZD at the start and 1 during treatment, respectively. Compared with aSTR, patients treated with oSTR had more grade 3-4 peripheral neuritis (9 (11.2%) vs. 2 (2.6%), p<0.001), grade 3–4 anaemia (12 (15.0%) vs. 3 (3.8%), p=0.002), and grade 3–4 arthralgia (1 (1.3%) vs. 0 (0.0%), p=0.002). Blood transfusion was given to 15 patients with severe anaemia: 12 on oSTR and 3 on aSTR. Two of whom died from the related severe AE: one oSTR and one aSTR. Compared with oSTR, patients treated with aSTR had more grade 1–2 ototoxicity (7 [9.0%]) vs. 0 [0.0%], p=0.006) and grade 1–2 nephrotoxicity (13 [16.7%] vs. 5 [6.2%], p=0.039). None progressed to grade 3 (Table 2). Patients treated with oSTR were at a 3-fold increased risk of having a grade 3–4 AE (aHR:3.04;95%CI:1.36–6.80) compared to patients treated with aSTR. The occurrence of grade 3-4 AE was also associated with TB clinic (Table 3).

**Table 2.** Patients experienced adverse events, by approach.

	Overall N = 158	oSTR N = 80	aSTR N = 78	p-value
**Any TB drug-related grade 3–4 AE**	34 (21.5%)	26 (32.5%)	8 (10.3%)	<0.001
**Number of grade 3–4 AE per patient**				<0.001
0	124 (78.5%)	54 (67.5%)	70 (89.7%)	
1	26 (16.5%)	20 (25.0%)	6 (7.7%)	
2	7 (4.4%)	6 (7.5%)	1 (1.3%)	
3	1 (0.6%)	0 (0.0%)	1 (1.3%)	
**Number of any grade AE per patient**				0.20
0	18 (11.4%)	6 (7.5%)	12 (15.4%)	
1	35 (22.2%)	17 (21.2%)	18 (23.1%)	
2	42 (26.6%)	19 (23.8%)	23 (29.5%)	
3	35 (22.2%)	20 (25.0%)	15 (19.2%)	
4	20 (12.7%)	12 (15.0%)	8 (10.3%)	
5	7 (4.4%)	6 (7.5%)	1 (1.3%)	
7	1 (0.6%)	0 (0.0%)	1 (1.3%)	
**Neurotoxicity**				<0.001
None	108 (68.4%)	43 (53.8%)	65 (83.3%)	
Grade 1–2	39 (24.7%)	28 (35.0%)	11 (14.1%)	
Grade 3–4**^A^**	11 (7.0%)	9 (11.2%)	2 (2.6%)	
**Anaemia**				0.002
None	101 (63.9%)	41 (51.2%)	60 (76.9%)	
Grade 1–2	42 (26.6%)	27 (33.8%)	15 (19.2%)	
Grade 3–4**^B^**	15 (9.5%)	12 (15.0%)	3 (3.8%)	
**Arthralgia**				0.002
None	141 (89.2%)	65 (81.2%)	76 (97.4%)	
Grade 1–2	16 (10.1%)	14 (17.5%)	2 (2.6%)	
Grade 3–4	1 (0.6%)	1 (1.3%)	0 (0.0%)	
**Ototoxicity**				0.006
None	151 (95.6%)	80 (100.0%)	71 (91.0%)	
Grade 1–2	7 (4.4%)	0 (0.0%)	7 (9.0%)	
**Nephrotoxicity**				0.04
None	140 (88.6%)	75 (93.8%)	65 (83.3%)	
Grade 1–2	18 (11.4%)	5 (6.2%)	13 (16.7%)	
**Hepatotoxicity**				0.07
None	94 (59.5%)	54 (67.5%)	40 (51.3%)	
Grade 1–2	57 (36.1%)	22 (27.5%)	35 (44.9%)	
Grade 3–4**^C^**	7 (4.4%)	4 (5.0%)	3 (3.8%)	
**Nausea/vomiting**				0.86
None	53 (33.5%)	26 (32.5%)	27 (34.6%)	
Grade 1–2	97 (61.4%)	49 (61.3%)	48 (61.5%)	
Grade 3–4	8 (5.1%)	5 (6.2%)	3 (3.8%)	
**QTC prolongation**				0.99
None	155 (98.1%)	78 (97.5%)	77 (98.7%)	
Grade 1–2	2 (1.3%)	1 (1.3%)	1 (1.3%)	
Grade 3–4	1 (0.6%)	1 (1.3%)	0 (0.0%)	
**Others AE^D^**				0.30
None	133 (84.2%)	65 (81.3%)	68 (87.2%)	
Grade 1–2	25 (15.8%)	15 (18.8%)	10 (12.8%)	

A Two patients who experienced grade 3-4 neurotoxicity in the aSTR were under linezolid (1 from the start of treatment and 1 after switching amikacin to linezolid).

B All 3 patients who experienced grade 3-4 anaemia in the aSTR were under linezolid from the start of treatment.

C The 3 patients who experienced grade 3-4 hepatotoxicity in the aSTR were under linezolid from the start of treatment.

D Others registered AE were gastritis disorders (19), skin rash/Pruritus (3), and ionic disturbances (3).

AE = adverse event; N = number; aSTR = Niger treatment strategy with a second-line injectable drug (SLID)-containing short treatment regimen (STR), with linezolid replacing the SLID in case of any grade of ototoxicity on monthly audiometry; oSTR = all oral short treatment regimen with linezolid throughout the intensive phase.

**Table 3.** Association between treatment approach and having a grade 3-4 adverse event, adjusted for potential confounders.

	Total	No grade 3–4 AE	Grade 3–4 AE				
	N	N (%)	N (%)	HR^A^	95%CI	aHR^A^	95%CI
**Total**	158	124 (78.5%)	34 (21.5%)				
**TB Clinic**							
Niamey	83	71 (85.5%)	12 (14.5%)	1		1	
Maradi	33	24 (72.7%)	9 (27.3%)	1.79	[0.75,4.28]	1.49	[0.62,3.58]
Tahoua	31	20 (64.5%)	11 (35.5%)	2.51*	[1.10,5.72]	2.29*	[1.01,5.21]
Zinder	11	9 (81.8%)	2 (18.2%)	1.18	[0.26,5.31]	0.91	[0.20,4.12]
**Gender**						NS	
Male	122	96 (78.7%)	26 (21.3%)	1			
Female	36	28 (77.8%)	8 (22.2%)	1.06	[0.48,2.34]		
**Age**						NS	
For every year increase in age	NA	NA	NA	1.02	[0.99,1.04]		
**HIV status^B^**						NS	
Negative	147	119 (81.0%)	28 (19.0%)	1			
Positive	9	5 (55.6%)	4 (44.4%)	2.35	[0.82,6.78]		
**Previous TB treatment**						NS	
No	38	29 (76.3%)	9 (23.7%)	1			
Yes	120	95 (79.2%)	25 (20.8%)	0.91	[0.42,1.98]		
**BMI**						NS	
For every increase in BMI unit	NA	NA	NA	1.02	[0.92,1.13]		
**Approach**							
aSTR	78	70 (89.7%)	8 (10.3%)	1		1	
oSTR	80	54 (67.5%)	26 (32.5%)	3.19**	[1.45,7.06]	3.04**	[1.36,6.80]

A Frailty bivariable and multivariable Cox regression models, accounting for the effect of randomisation month (clusters were defined by month of RR-TB diagnosis).

B 2 patients had HIV test results showing undetermined, category not shown. AE = adverse event; BMI = body mass index; aSTR = Niger treatment strategy with a second-line injectable drug (SLID)-containing short treatment regimen (STR), with linezolid replacing the SLID in case of any grade of ototoxicity on monthly audiometry; oSTR = all oral short treatment regimen with linezolid throughout the intensive phase; HR = hazard ratio; aHR = adjusted hazard ratio; NA = not applicable; NS = not significant;

* p < 0.05;

** p < 0.01.

### Treatment efficacy

With oSTR, 64 (81%) and 69 (87.3%) converted on smear microscopy by months 2 and 4, respectively, compared to 58 (74.4%) and 65 (83.3%) with aSTR. After excluding patients lost to follow-up (N=10) or with retrospectively identified initial FQ-resistance (N=1), 147 participants were included in the efficacy analysis (Figure 1). A favourable treatment outcome was achieved for 81.6 % (62/76) on oSTR compared to 78.9% (56/71) on aSTR. Unfavourable outcome (death or treatment failure) was registered in 14 (18.4%) patients on oSTR (11 deaths and 3 with treatment failure) compared to 15 (21.1%) patients on aSTR (15 deaths). Of three patients with treatment failure with oSTR approach, one patient died before DST results were known, and the remaining 2 patients were treated with a salvage regimen. These 2 patients were identified with frameshift mutations in *Rv0678* (insC: Asp47fs) by Deeplex Myc/TB® leading to an observed elevated minimal inhibitory concentration of 0.125 mg/L in 7H9 medium, which is commonly associated with borderline resistance to BDQ and clofazimine. No susceptibility testing was performed for BDQ or LZD at baseline, before RR-TB treatment initiation.

There was no statistically significant association (aOR:0.80;95%CI:0.18–3.65) between treatment outcome and treatment approach (Table 4). Having a clinically unfavourable outcome was significantly associated with TB clinic. A higher BMI at treatment initiation protected against having an adverse outcome (for every unit increase in BMI: aOR:0.76;95%CI:0.63–0.93).

**Table 4.** Association between treatment approach and having a clinically unfavourable outcome, adjusted for potential confounders.

	Total	Favourable outcome	Unfavourable outcomes				
	N	N (%)	N (%)	OR^A^	95%CI	aOR^A^	95%CI
**Total**	147	118 (80.3%)	29 (19.7%)				
**TB Clinic**							
Niamey	72	60 (83.3%)	12 (16.7%)	1		1	
Maradi	33	30 (90.9%)	3 (9.1%)	0.52	[0.12,2.19]	0.44	[0.09,2.10]
Tahoua	31	19 (61.3%)	12 (38.7%)	4.37*	[1.39,13.75]	5.45**	[1.57,18.88]
Zinder	11	9 (81.8%)	2 (18.2%)	1.23	[0.21,7.22]	1.67	[0.25,11.01]
**Gender**						NS	
Male	115	91 (79.1%)	24 (20.9%)	1			
Female	32	27 (84.4%)	5 (15.6%)	0.72	[0.24,2.15]		
**Age**						NS	
For every year increase in age	NA	NA	NA	1.03*	[1.00,1.06]		
**HIV status**						NS	
Negative	138	113 (81.9%)	25 (18.1%)	1			
Positive	7	4 (57.1%)	3 (42.9%)	4.19	[0.77,22.84]		
**Previous TB treatment**						NS	
No	34	28 (82.4%)	6 (17.6%)				
Yes	113	90 (79.6%)	23 (20.4%)	1.17	[0.41,3.32]		
**BMI**							
For every increase in BMI unit	NA	NA	NA	0.78*	[0.65,0.95]	0.76**	[0.63,0.93]
**Chest X-ray ^B^**						NS	
Normal	3	3 (100.0%)	0 (0.0%)	NA	NA		
Unilateral	6	5 (83.3%)	1 (16.7%)	1			
Unilateral & cavitary	12	10 (83.3%)	2 (16.7%)	1.05	[0.07,16.65]		
Bilateral	42	34 (81.0%)	8 (19.0%)	1.47	[0.13,16.20]		
Bilateral & cavitary	77	61 (79.2%)	16 (20.8%)	1.54	[0.15,15.84]		
**Bacillary load^B^**						NS	
Negative	11	9 (81.8%)	2 (18.2%)	1			
Scanty, 1+	43	38 (88.4%)	5 (11.6%)	0.75	[0.11,5.08]		
2+/3+	92	70 (76.1%)	22 (23.9%)	2.41	[0.41,14.35]		
**Approach**							
aSTR	71	56 (78.9%)	15 (21.1%)	1		1	
oSTR	76	62 (81.6%)	14 (18.4%)	0.84	[0.30,2.38]	0.80	[0.18,3.65]

A Multilevel bivariable and multivariable logistic regression models, accounting for the effect of randomisation month (clusters were defined by month of rifampicin-resistant TB diagnosis).

B Observations with missing values: 7 for chest X-ray, 1 for smear microscopy bacillary load. aSTR = Niger treatment strategy with a second-line injectable drug (SLID)-containing short treatment regimen (STR), with linezolid replacing the SLID in case of any grade of ototoxicity on monthly audiometry; oSTR = all oral short treatment regimen with linezolid throughout the intensive phase; NA = not applicable; NS = not significant;

* p < 0.05;

** p < 0.01.

Among 10 patients lost to follow-up, half (N=5) cited AEs and/or treatment duration as the reason, whereas the other half cited social reasons. Of 26 patients who died, 18 (69.2%) likely died because of advanced TB disease, including 2 patients with severe anaemia as AE (both on LZD, one during oSTR, one after switching from SLID to LZD in aSTR).

## DISCUSSION

This pragmatic clinical trial is the first to show that aSTR in Niger, in which LZD replaced the SLID in case of any grade of ototoxicity detected at baseline or during monthly audiometry, was safer than an oSTR treatment regimen with LZD throughout the intensive phase. Treatment efficacy was similar for both regimens. Regimen assignment by month of diagnosis was feasible and assured similar distribution of baseline characteristics between both arms.

AEs were reported in 88,6% of patients. Grade 3–4 AE observed included peripheral neuritis, anaemia, and arthralgia, which are known LZD-related effects.^1,6,17^ LZD-related AEs, like severe anaemia, can be life-threatening and challenging to manage in resource-limited settings. Soon, Niger will implement the new WHO-recommended 6-month BPaL/BPaLM regimen, extended to 9 months in case of delayed conversion. Given the extent of AEs observed with 4 to 6 months of LZD in our study, robust drug safety and monitoring systems will be essential for programmatic rollout.^13,18^ In this study, mild and moderate ototoxicity and nephrotoxicity related to amikacin were more common with aSTR. Monthly monitoring and early switching from amikacin to LZD prevented severe SLID-related AE.^10,11^ While not life-threatening, ototoxicity affects communication, quality of life, and may worsen TB-related stigma.^19,20^ Of the 8 patients experiencing severe AE on aSTR, 7 had LZD exposure (6 from baseline, 1 as amikacin replacement due to ototoxicity). Ideally, a safer alternative to LZD would replace amikacin in such cases. Effective RR-TB treatment requires not only shorter, oral treatments but also medicines with better safety profiles.^21^ We monitored patients for AE and in the aSTR arm the SLID was replaced in a timely manner. The ability to adapt the regimen based on tolerance issues may have mitigated SLID-related adverse effects effectively, underscoring that adapting a drug regimen in response to tolerance issues can reduce toxicity without compromising efficacy, even when using drugs (like injectables) traditionally considered less tolerable.

The oSTR and aSTR arms had similar efficacy. This contrasts with studies showing that BDQ-containing oSTR regimens improve treatment success and/or reduce the risk of mortality when compared with injectable-containing regimens.^2,22^ The high treatment success rates in both arms likely reflect a decade of effective programmatic management of drug-resistant TB with short regimens in Niger. However, success was lower than in the past in Niger,^10^ possibly due to worsened access given increased security threats.

Several limitations merit consideration. First, the levels of experience among clinicians in AE monitoring differed across clinics; more experienced clinicians might have been more proficient in reporting and managing AEs accurately. However, this potential bias was mitigated by enrolling patients from both treatment arms at each facility, while ensuring regular supervision and case discussions. Second, the trial design did not allow for individual-level randomisation. The monthly change in regimen allocation lacks the blinding of conventional randomisation, which could theoretically allow manipulation of care-seeking behaviour. However, the likelihood of such bias is minimal. Third, the lack of data on relapse, as post-treatment follow-up is not complete. Previous results showed that relapse is rare after successful treatment with aSTR.^12,23^ Also, enrolment occurred in Niger alone, which may limit generalisability. However, the trial was conducted in routine care, enrolling patients from a nationwide cohort, thus reflecting the reality of the National TB Programme in Niger. The attribution of AEs to specific drugs was done by a board of experts but remains a limitation. Finally, our trial was open-label and observation bias might have occurred, and data on pharmacokinetics were not collected.

## CONCLUSION

The aSTR arm, with LZD replacing the SLID upon detection of ototoxicity, was associated with fewer grade 3-4 AE than oSTR. In the aSTR, monthly monitoring ensured timely replacement of the SLID (amikacin) with LZD. Both arms achieved similar treatment efficacy. Our findings suggest that tailoring treatment based on individual tolerance can mitigate toxicity without compromising efficacy, even when using injectable drugs that are typically considered poorly tolerated.
